# The change of EMG during lifting a object from floor according to foot position

**DOI:** 10.1186/1757-1146-7-S1-A118

**Published:** 2014-04-08

**Authors:** Lee Han Suk, Kim Jun Hoo

**Affiliations:** 1 The Department of Physical Therapy, Eulji University, Jaseng Hospital of Korean Eastern Medicine, Korea

## 

The purpose of this study is to represent a basic data about the comparison between EMG of lumbar and leg by the change of foot position.

Ten women in their twenties volunteered for this study. They took a measurement change between EMG of lumbar and leg with 'pick up a object' according to the change in foot position. Foot position degree was 0 and 45 degree. We measured 3 times for each person and the order of foot position was random. The EMG measure instrument(TeleMyo DTS telementry system; Noraxon, USA) was used in the study.

The muscle activation of TA(tibialis anterior), VL(vastus lateralis), MG(medial gastrocnemius), IC(iliocostalis) were increased in 45 degree and there were significant difference in TA, VL, IC of right side between o and 45 degree but only VL of left side has a significant difference between o and 45 degree (p<0.05).

These finding suggest that muscle activation during pick up a object differs depending on foot angle. We believe that these difference should be considered when physical therapist educate the proper posture to patient

